# Diabetic Retinopathy Screening Using Telemedicine Tools: Pilot Study in Hungary

**DOI:** 10.1155/2016/4529824

**Published:** 2016-12-19

**Authors:** Dóra J. Eszes, Dóra J. Szabó, Greg Russell, Phil Kirby, Edit Paulik, László Nagymajtényi, Andrea Facskó, Morten C. Moe, Beáta É. Petrovski

**Affiliations:** ^1^Department of Public Health, University of Szeged, Szeged, Hungary; ^2^Department of Ophthalmology, Albert Szent-Györgyi Clinical Center, University of Szeged, Szeged, Hungary; ^3^Health Intelligence, Clinical Development, Chesire, UK; ^4^Centre of Eye Research, Department of Ophthalmology, Oslo University Hospital, University of Oslo, Oslo, Norway; ^5^Health Services Research Centre, Akershus University Hospital, Lørenskog, Norway; ^6^Institute of Clinical Medicine, University of Oslo, Campus Ahus, Oslo, Norway

## Abstract

*Introduction.* Diabetic retinopathy (DR) is a sight-threatening complication of diabetes. Telemedicine tools can prevent blindness. We aimed to investigate the patients' satisfaction when using such tools (fundus camera examination) and the effect of demographic and socioeconomic factors on participation in screening.* Methods.* Pilot study involving fundus camera screening and self-administered questionnaire on participants' experience during fundus examination (comfort, reliability, and future interest in participation), as well as demographic and socioeconomic factors was performed on 89 patients with known diabetes in Csongrád County, a southeastern region of Hungary.* Results.* Thirty percent of the patients had never participated in any ophthalmological screening, while 25.7% had DR of some grade based upon a standard fundus camera examination and UK-based DR grading protocol (Spectra™ software). Large majority of the patients were satisfied with the screening and found it reliable and acceptable to undertake examination under pupil dilation; 67.3% were willing to undergo nonmydriatic fundus camera examination again. There was a statistically significant relationship between economic activity, education and marital status, and future interest in participation.* Discussion.* Participants found digital retinal screening to be reliable and satisfactory. Telemedicine can be a strong tool, supporting eye care professionals and allowing for faster and more comfortable DR screening.

## 1. Introduction

The global incidence of diabetes mellitus (DM) among adults (age 18 years and older) was 9% worldwide in 2014 [1], while its prevalence still shows an increasing tendency due to obvious obesity epidemic and aging of the population [2–4]. In Hungary, a total of 865 069 patients (9.5% of the population) suffered from DM in the same age group in 2011 [5], and some degree of diabetic retinopathy (DR) could be observed among 19% of the patients with type 1 DM (T1DM) and 24% in those suffering from type 2 DM (T2DM) for 3 or 4 years [6]. DR is the fourth most common cause of blindness in the overall population, but it is in second place among active adults in industrialized countries [7], accounting for a significant drop in quality of life (QoF) and working ability of the patients [8, 9]. In a study comparing data from 35 populations, the global prevalence of sight-threatening retinopathy (STR) was estimated at 10.2% for all DM patients [10]. Known risk factors for developing DR are age, gender, duration, and type of DM, elevated HbA_1_c, high blood pressure, and retinopathy stage, while other correlating risk factors are being investigated. Unfortunately, 50% of the people with diabetes are unaware of the characteristics of their disease and the compliance in attending screening programs is poor. The disease is determined by the outcome of the complications. Since high blood sugar and fat destroy the wall of the arteries, it is not surprising that people with diabetes have 2 to 4 times higher cardiovascular mortality rate and 2 to 4 times higher risk of strokes than patients without diabetes. Renal failure is also a common complication with the estimated number of 30–40% of the patients with diabetes, while 60–70% of the patients develop neuropathy. This is not only an individual problem, but a societal problem as well. According to a 2009 survey, the average annual health expenditure for diabetic patients was $1205 per capita and for patients with complications this number was $2276 per capita. Half of this cost is made up of drugs, but only a quarter of the cost spent on drugs is for antidiabetics [11]. Similarly, the treating expenses doubled in Germany and America, where $174 billion was spent on the treatment of diabetes in 2007 [11]. The Hungarian data cover only the cost of the National Health Insurance Fund, while there are other economic aspects like time off from work or restricted work due to complications of the disease. DR is caused by damage to the retinal microvasculature. Proper screening for DR is an important milestone towards achieving early and efficient laser photocoagulation and/or anti-vascular endothelial growth factor (anti-VEGF) treatment for preventing visual loss [12]. Depending on the severity of DR, four stages can be distinguished in general: preretinopathy (R0), background retinopathy (R1), preproliferative retinopathy (R2), and active proliferative retinopathy (R3A) [13]. A further subclassification exists for stable proliferative retinopathy (R3S) in patients who have received panretinal laser photocoagulation (PRP) under R3A and then became “stable”; these cases are considered safe to keep in a surveillance clinic [14]. Once fundus lesions appear as a complication of DM, the patient has an apparent DR with either low, intermediate, or high risk for developing some grade of DR. Therefore, the focus should rather be on raising prevention programs and early detection, as well as successful treatment of the basic disease.

DR is usually asymptomatic before the appearance of any vision loss, but it is detectable by retinal imaging techniques objectively and by accurately taken best corrected VA measurements. Much research around the world has been focused on the use of telemedicine tools for fundus imaging and screening, the UK system standing up at the top in terms of reliability, precision, and standardized input and output. The results so far have been very promising, with each study being reported to date pointing out the high sensitivity for detecting several fundus lesions in the initial stages of DR by a standard fundus camera and a grading software [15].

The Spectra DR software is designed around the requirements of the UK National Health Service (NHS) national screening program for DR; it is highly complex and requires a high level of sophistication in the software to meet its requirements. Spectra DR enables patient appointments to be created, data entry, image capture, and grading. A generation of patient results is provided together with a report regarding the patients' screening prediction via a “plug-in” algorithm. With the use of nonmydriatic or investigational hand-held portable cameras, a quick and simple DR evaluation process will likely improve the patients' willingness to participate in future screening tests.

In 1980, Iceland began regular DR screening for T1DM patients, which resulted in the reduction of disease-related blindness from 2.4% to 0.5% [16]. The Icelandic population being used for the cohort study and development of a commonly used risk calculator (Risk Medical Solutions, Iceland) is much more homogenous when it comes to ethnic and socioeconomic differences compared to the population in Hungary. Nevertheless, with these new screening and telemedicine tools, it is realistic to expect similar results to be achieved in other European countries, including Hungary, within 5 to 10 years time [17].

The present research explores how DM patients subjectively experience the telemedicine tools and examination through participation in a free fundus camera screening program conducted in a southeastern county (Csongrád) in Hungary and obtains feedback on whether they would participate in such an examination in the future. Furthermore, demographic factors such as age, gender, economic activity, and socioeconomic status (SES) (level of education, support from family, and subjectively perceived financial status) are examined for their effect upon participation in future screening programs.

## 2. Methods

A free screening test was performed on a random population including 178 eyes from 89 patients with confirmed DM diagnosis. Handling of the fundus camera and the image acquisitions were performed by a qualified professional in a darkened room, which were then forwarded through a secure internet connection to a specialist doctor/ophthalmologist (A. F./M. C. M.) or ROG (G. R./P. K.) for evaluation. In case of constricted pupil, another image was taken after ensuring normal intraocular pressure level and applying cyclopentolate (5 mg/mL) eye drops to achieve mydriasis. The assessment of the fundus images was performed within 10 working days using Spectra DR software. The recordings were safely deposited and kept inaccessible to third parties for 10 years at a central server, so that later they can be used in further comparative studies on DR.

The images were acquired by an 18-megapixel Canon EOS digital camera which was connected to a Canon CR2 color, nonmydriatic, 45° retinal camera. Two pictures were taken of the participants' each eye: one with the macula and another with the optic nerve in the center—this is in line with the UK screening requirements [18]. In case of presence of amblyopia or nontransparent media (e.g. cataract and corneal or visual axis obstructing conditions), the patients were excluded from the study. During image evaluation, the graders (A. F./M. C. M./G. R./P. K.) classified the signs and the stages of DR and maculopathy in the standardized UK-based software Spectra DR and graded the images in alignment with the UK standard grading protocols [19]. Each image was evaluated in two stages: first, the ROG (G. R./P. R.) evaluated them, and then a supervisor/ophthalmic consultant confirmed the diagnosis (A. F./M. C. M.). At the end, an expert opinion regarding the grade of retinopathy was sent back to the screening site, that is, stage of retinopathy (R0/1/2/3A/S) and absence or existence of maculopathy (M0/1). Other discovered abnormalities were not diagnosed in this study, although they were recorded, as they can provide further information about other symptoms which may have occurred in the past, and therefore may require medical attention over a specified period of time.

The classification of the DR was as follows:

M0: no maculopathy was detected; repeated screening was recommended one year later.
M1: there was a sight-threatening maculopathy; within one month a medical examination is required.
R0: there was no clinical anomaly; repeated screening was recommended one year later.
R1: mild nonproliferative phase, microaneurisms, dot- or blot-like hemorrhages, or exudates could be seen; control examination was recommended one year later.
R2: moderate or severe nonproliferative phase, major bleeding(s), cotton-wool spots, venous looping, and intraretinal microvascular abnormalities (IRMAs) were visible; control examination was required within one month.
R3A: active proliferative phase, neovascularization of the optic disc (NVD) or elsewhere (NVE) or preretinal bleeding(s), vitreous bleeding, preretinal fibrosis, and tractional retinal detachment could be observed; immediate medical examination was required within two weeks.
R3S: stable proliferative retinopathy; a retinal image showing stable post-PRP laser with no signs or reactivation or active referable retinopathy; only to be determined in the presence of “benchmark images” taken at the time of discharge for comparison; screening intervals may be at the discretion of the trained ROG.

Other recorded, but not reported, changes/fundus pathology included age-related macular degeneration (AMD), glaucoma changes in the optic nerve, and any other signs of eye disease.

### 2.1. Self-Completed Questionnaire

The self-completed questionnaire collected information about the individual's demographic status such as age, gender, economic activity (full-time, part-time, and retired), SES such as education (primary, secondary, and higher), and marital status (married or lives with a partner, single, separated or divorced, and widowed). The general part of the questionnaire was based on the European Health Interview Survey 2009 [20], and it collected data about DR associated exposure parameters and some other health connected parameters, type of DM, or presence of hypertension, as well as the type of eye diseases. Furthermore, data were collected about the frequency of measuring blood sugar levels and also about participation in screening programs, which are important for preventing retinopathy, including the frequency of attending Diabetology or Ophthalmology specialist clinics. Questions regarding the perceived reliability of results (yes/no/maybe), willingness to participate again (yes/no/maybe), comfortability (dissatisfied/satisfied/acceptable) of the tests performed, and the overall perception of the screening examinations as well as whether they would participate in a similar examination next time were being asked/collected as well. Some categories underwent merging due to missing data, for example, the intensity of blood sugar measurement (monthly/less than a month, weekly/every few days, and daily/more than once a day). If the participants could not understand or read the questionnaire for whatever reason, they received professional help accordingly.

### 2.2. Statistical Analysis

The analysis of the data was performed by descriptive statistical analysis on *N* number of participants, and percent distribution, median, and interquartile range (IQR) are being shown. The Chi-square (*χ* ^2^) and Fisher exact tests were used to test differences of the distributions of categorical variables. The relationship between two variables was considered statistically significant when *P* < 0.05. The graphs were made in GraphPad Prism 5.01 (GraphPad Software Inc., La Jolla, CA, USA). The statistical analysis of the data was performed by using Stata (Intercooled Stata 8.0, Stata Corporation, College Station, TX, USA) and Excel software (Microsoft Corporation, USA).

### 2.3. Ethical Issues

The Regional and Institutional Human Medical Biological Research Ethics Committee of the Albert Szent-Györgyi Health Centre, University of Szeged, approved the study protocol (number 197/2015). The research provided anonymity to the participants. Before the beginning of a test, the participants signed a written consent form in which they agreed to permit the use of data for research purposes.

## 3. Results

**Figure 1:**
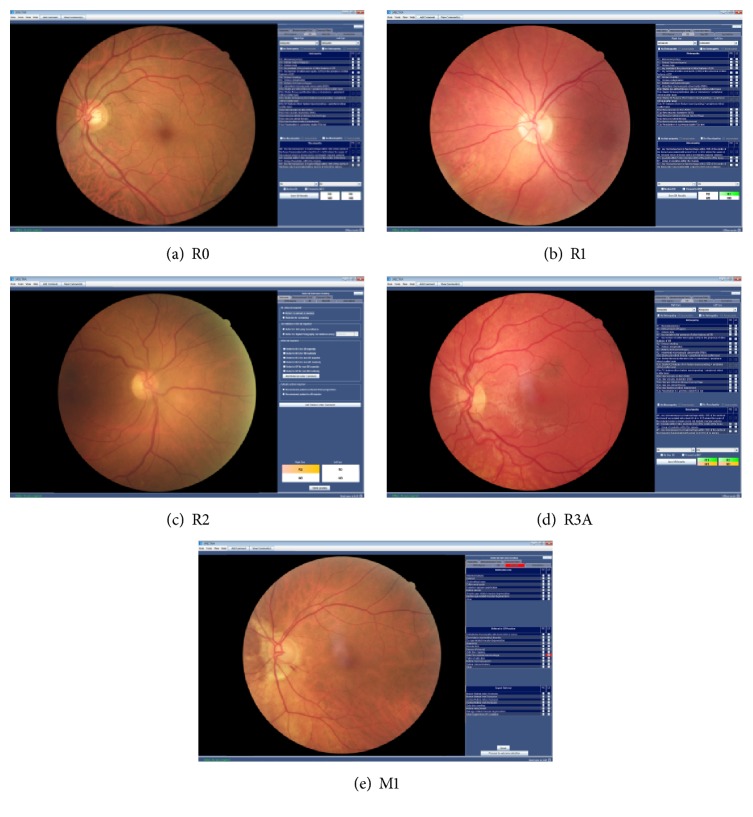
Spectra DR based grading of the DR retinopathy. Representative images of the different grading stages are shown in the studied population.

178 eyes of 89 people were examined in the study out of which 30 were men (33.7%) and 59 were women (66.3%).  Table 1 shows the demographic characteristics of the patients, the median age of whom ranged between 56 and 68 years of age and had median HbA1c of 7.2% (ranging between 6.4 and 7.9%).  Table 2 shows the distribution of the types of DM and the stages of DR in the screened population, based upon and compared to the UK grading system and software (Spectra DR). Twenty percent of the participants had T1DM out of which 70.8% had T1DM diagnosed by a Diabetology department, the rest being yet undiagnosed or hidden disease patients. Mild nonproliferative DR (grade R1) was detected in 23.0% of the participants, while higher (moderate/R2 and proliferative/R3) grade DR was detected in 1.4% and 1.4% of the subjects, respectively; maculopathy/M1 was present in 5.4% of the studied group (representative images from these were captured from each grade and processed in the Spectra software as shown in Figure 1). Another retinal pathology was detected in 28.4% of the participants. There was an overall left-shift in the distribution of earlier stages of DR in the UK population compared to the one represented by the Hungarian graded images and based upon the Spectra DR software, probably due to the existence of a well established screening system in the UK and early detection of the disease.

According to the self-perceived satisfaction with the classical pupil dilation versus fundus camera examination, 20.4% versus 83.6% of the participants expressed satisfaction, respectively, while 37.0% versus 9.1% were unsatisfied, and 42.6% versus 7.3% could not decide. The classical pupil dilation versus fundus camera examination was found to be definitely reliable by 75.5% versus 72.0%, possibly reliable by 18.4% versus 16.0%, and unreliable by 6.1% versus 12.0%, respectively. The willingness to participate in a classical pupil dilation versus fundus camera examination was found to be positive by 78.2% versus 67.3%, while 9.1% versus 10.9% responded that they would not participate, and 12.7% versus 21.8% responded as maybe doing it. There was no significant difference between the satisfaction from the examination (*P* = 0.9) and reliability (*P* = 0.3), although the willingness to participate significantly differed between the classical versus fundus camera examination (*P* = 0.01) (Table 3).

**Figure 2:**
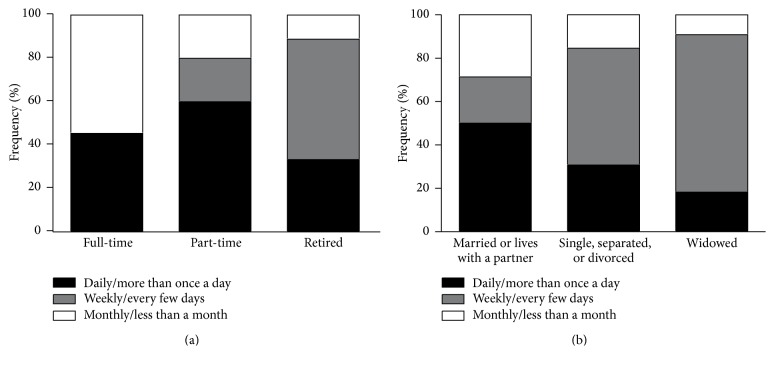
Effect of economic activity (a) and marital status (b) upon the blood sugar screening frequency.

The economic activity significantly affected the participation in a blood sugar screening (*P* = 0.001). Sixty percent of those employed in a part-time job had attended blood sugar screening more than once a day or daily, 20% weekly/every few days or monthly/less than a month. The daily/more than once a day attendance was 33.3% among retired, while the weekly/every few days screening was 55.6%, and the monthly/more than a month was 11.1% in this age/patient group. Among the full-time workers, the daily/more than once a day and monthly/less than a month screening was 45.5% versus 54.5% (Figure 2(a)). Similarly, marital status (being married or living with a partner) significantly impacted the likeliness to attend blood sugar screening (*P* = 0.04); this population had a higher daily/more than once a day blood sugar screening attendance, with a frequency of 50% compared to those living alone (single, separated, or divorced: 30.8%; widowed: 18.2%); the latter two populations had otherwise the highest weekly/every few days attendance (single, separated, or divorced: 53.9%; widowed: 72.7%). The least frequent or monthly/less than a month screening attendance was the highest among married or living with a partner population (28.6%), while it was the smallest among widowed participants (9.1%) (Figure 2(b)).

**Figure 3:**
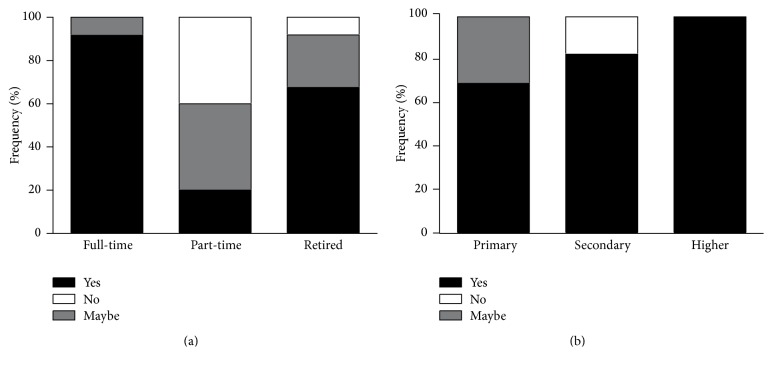
Economic activity in relation to willingness (a) and educational level in relation to satisfaction (b) in participating in fundus screening examination.

The willingness to participate in the annual fundus camera screening was the highest among the full-time workers (91.7%) and the lowest among part-time workers (20.0%) Those who reported maybe versus no attendance were higher among part-time workers (40.0% versus 40.0%, resp.), while the willingness to participate differed significantly between the analyzed economical groups (*P* = 0.003) (Figure 3(a)). The satisfaction with the fundus camera screening also increased significantly with the level of education (primary (69.2%) and secondary (82.8%); higher (100%), *P* = 0.003) (Figure 3(b)).

Among participants with secondary or higher education, the most common argument used against the classical fundus exam was “I cannot see clearly after.” The participants with primary school level education had significantly higher rate of stating dissatisfaction of the pupil dilation examination. This reason was not stated among higher educated patients, although the “I cannot drive after” reason seemed to appear more often in this group of patients.

## 4. Discussion

The present study aimed to investigate the patients' experience with the use of telemedicinal tools for screening of DR and the ability to collect the parameters needed to calculate DR risk (age, gender, type and duration of DM, HbA1c, hypertension, and fundus image grading) in a southeastern county (Csongrád) in Hungary. The justification for using health care tools aimed at screening DR is high, due to the great availability of tools for DR prevention and avoidance of late complications such as STR. The population of Csongrád County is very plausible for initiating such a study, since it has a known higher prevalence of DM compared to other counties in Hungary [5]. In addition, the study followed the progressive trend of DM worldwide and examined the willingness to participate in screening tests, the attitude towards screening examination, and the influence of demographics and socioeconomic factors like education, financial, and marital status. Regarding the risk factors, the SES has been already shown to have a very significant impact on the attendance in screening examinations, while occupation has been related to a greater impact on nonattendance in screenings [21]. The screening frequency for blood sugar levels in full-time workers was indeed significantly lower in our study, but the willingness to participate in fundus screening examination was higher in that subpopulation.

From the standpoint of DR formation and progression, it is 76.4% of the patients who had high blood pressure which, by itself or as a codisease, gives poorer prognosis for the DM patients due to a predisposition for premature vascular sclerosis. The occurrence of DR in the studied sample population was 25.5%, which is higher than any previous results in Hungary [22], although somewhat expected in Csongrád County.

Although the Diabetology guidelines recommend blood glucose levels to be checked several times a day, only a little over a quarter of the participants performed it accordingly. Strikingly, 60% of the study participants performed blood glucose testing every few days, if not more rarely. Upon diagnosis with DM, the Diabetologist or the General Practitioner informed the patient of the possible complications from the disease and recommended an annual eye screening test. Our results coincide with the International Diabetes Federation's (IDF's) observation that 50% of the people with DM are not aware of the characteristics of their disease [23]. In Hungary, the number of known patients with diabetes makes nearly 10% of the total population. It would take 100 ophthalmologists (from the total of 968 practicing) working full-time if they want to carry out only the annual screenings by using traditional tools on such a sized population. This may change by using the telemedicine system [17]. Introducing a new screening program always faces challenges, but previous studies from other countries show promising results. DR could be detected at early stages by digital imaging even in rural areas [24]. Diabetes causing vision loss is successfully confined in countries like Iceland, where regular screening was implemented.

In our study, only a third of the participants had not visited an ophthalmologist, while 12.4% of them have been diagnosed with DM within a year; only 56.2% of the participants complied with the one-year recommendation. In the UK, patients compliance in attending traditional screening was 45% and 50% in fundus camera screening in the first year [25]. After using a mobile fundus camera screening unit to reach more patients, the compliance elevated to 80% in the fifth year [26]. Compliance is a highly influential factor of cost effectiveness because of the fixed costs (digital imaging camera, computer system, etc.) [25]. Patient satisfaction affects the attendance rate of the screening. The response to the subjective experiences perceived during fundus examination did produce satisfactory results: more than three-quarters of the participants were satisfied with the fundus camera examination and one out of five with the traditional method. In both cases, three-quarters of the participants considered the results of the study to be reliable, a significant difference being found between the two screening procedures. There were fewer problems than expected (e.g., subjects being not able to drive after pupil dilation), but it can be a factor which is most likely related to older age of the sampled population. It is interesting to note, however, that during the procedure of pupil dilation, one quarter of the subjects found administering eye drops being irritating or uncomfortable, in particular, those who had lower education.

There is a level of contradiction in the assessment of reliability and satisfaction in the study, since significantly more people were willing to participate in the traditional retinal screening method than in the fundus camera test (78.2% versus 67.3%). A possible weakness of the study is the size of the sample. 83.6% of the participants were dissatisfied with the examination, which raises the suspicion they could have chosen “Other” for their response to having no other comments, and this could have been done out of necessity. Among the inconvenience experienced during the test with pupil dilation, the “Other” category was chosen by only 4.1% in which no mention of any reasons for the selection made was stated whatsoever.

During the analysis, the economic activity and education appeared to pose an effect on the individual's willingness to participate in the screening test. The fundus camera test was preferred mostly by the full-time employees, with whom it was presumably important to see well after the test in order to be able to continue their work during the same day. Based on the level of education, the few subjects that evaluated the fundus camera test as satisfactory were those who found eye drops to be the most uncomfortable in the traditional test. These data are somewhat contradictory, as mydriatic drops are always required in traditional testing. People with higher education found only the driving restriction and the bad sight after the examination as a negative aspect of the screening; in this context, they were 100% satisfied with the fundus camera test.

The telemedicine part of the study also concerns data safety and patient anonymity preservation which are now guided by an EU law contained in the Charter of Fundamental Rights of the European Union, Article 8 (2000/C 364/01) [27], as well as the need to safely store and make backup files for high resolution fundus images acquired from the patients and their retention; these rules were followed in the present study entirely. The issue of having decentralized and near the patient DR screening and fundus imaging services and centralized image reading remains to be evidenced in future telemedicinal studies for screening DR in Hungary, the UK grading system being the golden standard for achieving the task properly.

In conclusion, the analyzed demographic and socioeconomic factors showed a significant relationship with the future participation in the fundus camera screening for DR. The participants' age or gender appears not to affect the experience (satisfaction) of the examination (e.g., fundus examination under pupil dilation). However, the level of education appears to have an important role: higher educated patients were more likely to participate in pupil dilation examination using an ophthalmoscope. This is in contradiction to the fact that only slightly more than half of the participants in this group took part in such screening examination within a period of one year. It was also not confirmed that the distribution of DR grades in this study is similar to the results of previous national studies [17, 22], as Csongrád County is not a representative population comparable to other parts of Hungary where the prevalence of DM and DR is lower. Further research is therefore needed on a larger or more representative sample from different counties in Hungary where the percent of distribution of patients diagnosed with DM varies.

In general, the treatment of DM patients is an interdisciplinary task of primary care physicians, diabetologists/dietologists, ophthalmologists/optometrists, and public health specialists. These professionals are responsible for giving lifestyle advice and for directing patients towards more appropriate screening tests. Ophthalmic monitoring is required every year after the diagnosis of diabetes and every other year for patients with excellent glycemic control without retinopathy at the previous examination but annually if there are risk factors [28]. Furthermore, if retinopathy is manifested to some degree, the screening time should be reduced to half a year (in the case of nonproliferative retinopathy) and three months (for preproliferative retinopathy). In case of proliferative retinopathy patients should go immediately to an ophthalmologist, in order to initiate laser treatment in time and thus save the eye from STR. The present state, unfortunately, seems to involve lack of realistic assessment or judgment of the risk from complications by the patients, and therefore a neglect to participate in the recommended screening tests. Constant maintenance of normal blood sugar levels is indispensable. Fast, easy, and accurate fundus camera examination is an alternative to the traditional, time-consuming, and “unsatisfactory” fundus test under pupil dilation. The patients, who tried this method, agreed that this new way is more satisfaction than the one they got used to, while they appreciated its reliability in the same way. Indeed, in the UK, due to the systematic screening implemented, DR is no longer the leading course of blindness in the working age population [29].

## Figures and Tables

**Table 1:** Patients' demographics.

Variables	Percent (%)
Gender	
Male	33.7%
Female	66.3%
Age (median, IQR)	63 (56–68)
HbA1c (median, IQR)	7.2 (6.4–7.9)
Hypertension ^*∗*^	
No	19.1%
Yes	76.4%
Occupation	
Full-time	22.2%
Part-time	9.3%
Retired	68.5%
Education ^*∗*^	
Primary	23.6%
Secondary	52.3%
Higher	21.8%
Marital status	
Married or lives with a partner	55.6%
Single, separated, or divorced	24.1%
Widowed	20.4%
Attendance of blood sugar screening	
Monthly/less than a month	20.8%
Weekly/every few days	39.6%
Daily/more, than once a day	39.6%

IQR: interquartile range.

^*∗*^The remaining percent of participants either were not aware of their disease (hypertension) or provided no response (education).

**Table 2:** Distribution of the DM types and DR grade in the studied population in relation to the UK-based grading system implemented by the Spectra™ analysis.

	T1DM	T2DM	R0	R1	R2	R3	M1
Csongrád County, Hungary	20.2%	79.8%	73.0%	23.0%	1.4%	1.4%	5.4%
East Anglia, UK	15.0%	85.0%	68.7%	27.5%	0.6%	0.3%	3.2%

**Table 3:** Reliability, satisfaction, and willingness to participate again in a classical or fundus camera examination for DR screening.

Variables	Pupil dilation	Fundus camera	*P* ^*∗*^
	*N* = 89	*N* = 89	
	(%)	(%)	
Reliability of the examination			
Yes	75.5%	72.0%	0.3
No	6.1%	12.0%	
Maybe	18.4%	16.0%	
Willingness to participate again			
Yes	78.2%	67.3%	0.01^*∗*^
No	9.1%	10.9%	
Maybe	12.7%	21.8%	
Satisfaction with the comfort of the screening			
Dissatisfied	37.0%	9.1%	0.9
Satisfied	20.4%	83.6%	
Acceptable	42.6%	7.3%	

^*∗*^ *P* < 0.05.

## References

[1] WHO http://www.who.int/gho/en/.

[2] Danaei G., Finucane M. M., Lu Y. (2011). National, regional, and global trends in fasting plasma glucose and diabetes prevalence since 1980: systematic analysis of health examination surveys and epidemiological studies with 370 country-years and 2·7 million participants. *The Lancet*.

[3] Saaddine J. B., Honeycutt A. A., Narayan K. M. V., Zhang X., Klein R., Boyle J. P. (2008). Projection of diabetic retinopathy and other major eye diseases among people with diabetes mellitus: United States, 2005–2050. *Archives of Ophthalmology*.

[4] World Health Organization Global report on diabetes. http://apps.who.int/iris/bitstream/10665/204871/1/9789241565257_eng.pdf?ua=1.

[5] Yearbook H. S. http://www.ksh.hu/docs/hun/xftp/idoszaki/evkonyv/evkonyv_2011.pdf.

[6] Németh J., Frigyik A., Vastag O. (2005). Causes of blindness in Hungary 1996–2000. *Ophthalmologia Hungarica*.

[7] WHO Prevention of blindness and visual impairment. http://www.who.int/blindness/causes/en/.

[8] Langelaan M., De Boer M. R., Van Nispen R. M. A., Wouters B., Moll A. C., Van Rens G. H. M. B. (2007). Impact of visual impairment on quality of life: a comparison with quality of life in the general population and with other chronic conditions. *Ophthalmic Epidemiology*.

[9] Jones S., Edwards R. T. (2010). Diabetic retinopathy screening: a systematic review of the economic evidence. *Diabetic Medicine*.

[10] Yau J. W. Y., Rogers S. L., Kawasaki R. (2012). Global prevalence and major risk factors of diabetic retinopathy. *Diabetes Care*.

[11] Vokó Z., Nagyjánosi L., Kaló Z. (2009). Direct health care costs of diabetes mellitus in Hungary. *LAM*.

[12] ETDRS (1991). Early photocoagulation for diabetic retinopathy. ETDRS report number 9. Early Treatment Diabetic Retinopathy Study Research Group. *Ophthalmology*.

[13] Government U. Diabetic eye screening: retinal image grading criteria. https://www.gov.uk/government/publications/diabetic-eye-screening-retinal-image-grading-criteria.

[14] Government U. Diabetic eye screening: surveillance pathways. https://www.gov.uk/government/publications/diabetic-eye-screening-surveillance-pathways.

[15] Lund S. H., Aspelund T., Kirby P. (2016). Individualised risk assessment for diabetic retinopathy and optimisation of screening intervals: a scientific approach to reducing healthcare costs. *The British Journal of Ophthalmology*.

[16] Stefánsson E., Bek T., Porta M., Larsen N., Kristinsson J. K., Agardh E. (2000). Screening and prevention of diabetic blindness. *Acta Ophthalmologica Scandinavica*.

[17] Somfai G., Ferencz M., Fiedler O., Varga T., Somogyi A., Németh J. (2007). Diabetic retinopathy at the beginning of the 21th century: prevention, diagnostics and therapy. *Magyar Belorvosi Archivum*.

[18] Government U https://www.gov.uk/government/publications/diabetic-eye-screening-pathway-for-images-and-where-images-cannot-be-taken.

[19] Government U. Diabetic eye screening: assuring the quality of grading. https://www.gov.uk/government/publications/diabetic-eye-screening-assuring-the-quality-of-grading.

[20] European Health Interview Survey 2009. http://www.ksh.gov.hu/elef/archiv/2009/pdf/elef_kerdoiv_alap.pdf.

[21] Zackrisson S., Andersson I., Manjer J., Janzon L. (2004). Non-attendance in breast cancer screening is associated with unfavourable socio-economic circumstances and advanced carcinoma. *International Journal of Cancer*.

[22] Fiedler O., Hargitai Z., Bíró Z. (2010). Telemedical diabetic retinopathy screening. Hungarian pilot study. *Magyar Belorvosi Archivum*.

[23] Federation ID http://www.idf.org/worlddiabetesday/toolkit/gp/facts-figures.

[24] Zimmer-Galler I. E., Kimura A. E., Gupta S. (2015). Diabetic retinopathy screening and the use of telemedicine. *Current Opinion in Ophthalmology*.

[25] Tu K. L., Palimar P., Sen S., Mathew P., Khaleeli A. (2004). Comparison of optometry vs digital photography screening for diabetic retinopathy in a single district. *Eye*.

[26] Harding S. P., Broadbent D. M., Neoh C., White M. C., Vora J. (1995). Sensitivity and specificity of photography and direct ophthalmoscopy in screening for sight threatening eye disease: the Liverpool Diabetic Eye Study. *British Medical Journal*.

[27] Union E. Article 8. http://ec.europa.eu/health/data_collection/data_protection/in_eu/index_en.htm.

[28] Organization WH Prevention of blindness from diabetes mellitus. http://www.who.int/blindness/causes/PreventionofBlindnessfromDiabetesMellituswithcoversmall.pdf?ua=1.

[29] Liew G., Michaelides M., Bunce C. (2014). A comparison of the causes of blindness certifications in England and Wales in working age adults (16–64 years), 1999-2000 with 2009-2010. *BMJ Open*.

